# Reduced graphene oxide modulates antioxidant responses and specialized metabolism in the medicinal orchid *Dendrobium chrysotoxum*: an integrated transcriptomic and metabolomic perspective

**DOI:** 10.3389/fpls.2026.1912555

**Published:** 2026-07-21

**Authors:** Zhengenrong Deng, Meng Zhao, Aoxue Luo, Yijun Fan, Dingying Ye

**Affiliations:** Sichuan Agricultural University, Chengdu, China

**Keywords:** *Dendrobium chrysotoxum*, flavonoid metabolism, integrated transcriptomic and metabolomic analyses, reduced graphene oxide, terpenoid metabolism

## Abstract

*Dendrobium chrysotoxum* is an important medicinal orchid containing a wide range of bioactive compounds, including alkaloids, flavonoids, terpenoids, and bibenzyls. Despite its pharmacological importance, the physiological and molecular responses of this species to nanomaterial elicitation remain unclear. Reduced graphene oxide (rGO) has emerged as a potential nano-elicitor that can affect plant physiological status, antioxidant regulation, and specialized metabolism. To examine its effects on *Dendrobium chrysotoxum*, seedlings were grown under greenhouse conditions and exposed to a series of rGO concentrations. Changes in growth-related traits, antioxidant defense, polysaccharide accumulation, total flavonoid content, and transcriptomic and metabolomic profiles were assessed. The responses were strongly concentration- and time-dependent. At 21 d after treatment, 100 mg·L^−1^ rGO (T3) increased CAT, SOD, and POD activities by 225.2%, 387.8%, and 22.2%, respectively, compared with CK. Polysaccharide and soluble sugar contents were also increased by 206.7% and 35.0%, respectively. Leaf total flavonoid content showed a transient middle-stage increase, with T3 showing a 56.79% increase over CK at 14 d. Although MDA accumulation increased under rGO exposure, the MDA level in T3 at 21 d was 57.4% and 71.3% lower than those in T4 and T5, respectively, indicating lower membrane lipid peroxidation than under high-dose treatments. Multi-omics analysis further detected 1,804 DEGs and 1,005 differential metabolic features with putative annotations, with flavonoid and terpenoid-related metabolism emerging as two major pathways altered by rGO exposure. Within these pathways, candidate genes encoding glycosyltransferases, cytochrome P450s, and sesquiterpene synthases, along with regulatory factors such as DcMYB20 and DcbHLH130, were associated with treatment-related variation in flavonoid and terpenoid metabolism. These results clarify the response pattern of a medicinal orchid to rGO treatment and provide a basis for future studies on nano-elicitation responses and candidate metabolic regulation in *Dendrobium*.

## 1 Introduction

Nanomaterials (NMs) are increasingly regarded as a novel class of abiotic nano-elicitors. When applied at subtoxic levels, they can induce stress-related signaling, activate defense priming, and modulate reactive oxygen species (ROS) homeostasis, thereby reshaping specialized metabolic networks and influencing the biosynthesis and accumulation of secondary metabolites in plants (Anjum et al., 2019). Among carbon-based nanomaterials, graphene has attracted sustained attention because of its distinctive physicochemical properties (Sharma et al., 2025), including high electrical conductivity, mechanical strength, thermal conductivity, and optical transmittance (Avouris, 2010). Previous studies have shown that graphene can promote seed germination when supplied at appropriate concentrations (Pandey et al., 2019). Reduced graphene oxide (rGO), a partially deoxygenated derivative of graphene oxide (GO), retains the *sp*^2^ carbon framework and a proportion of oxygen-containing functional groups, giving it favorable electrical conductivity, strong surface activity, and high adsorption and loading capacity. These features have made rGO attractive for a range of applications, including serving as slow-release fertilizer carriers (El-Sakhawy et al., 2025), promoting plant growth (Sharma et al., 2025), enhancing antibacterial performance, improving plant stress tolerance (Facure et al., 2020), increasing germination rates, regulating root development, enhancing resistance to environmental stress, and alleviating oxidative stress in plants (Dutta et al., 2022). In addition, owing to its large specific surface area and active interfaces, rGO can influence ROS dynamics and coordinate antioxidant enzyme responses (Zhang et al., 2022). For example, carbon nanomaterials and carbon nanotubes have been reported to mediate nanopriming, mimic natural mechanical stimulation, and activate innate immune defense systems in plants (Cui et al., 2024). Despite this potential, whether rGO exerts stable and beneficial effects on the cultivation and production of medicinal plants has not yet been clearly established.

*Dendrobium* is a medicinally important genus within the Orchidaceae family and is valued for its rich repertoire of bioactive constituents such as alkaloids, polyphenols, flavonoids, terpenoids, and biphenyls (He et al., 2020; Nie et al., 2020). It has high medicinal and developmental value (Wang et al., 2010). Within this genus, *D.chrysotoxum* is recognized as an important medicinal resource because of its abundant secondary metabolites (Li et al., 2022). However, the accumulation of these active compounds is closely related to plant growth status, environmental stress, and cultivation practices. Therefore, a major challenge is to understand how cultivation practices influence plant growth and specialized metabolism without causing excessive stress or disrupting normal growth. This highlights the practical value of developing regulation strategies that are efficient, economical, and environmentally sustainable.

To address this issue, the present study focused on the physiological responses, metabolic reprogramming, and molecular regulatory features of *D.chrysotoxum* under rGO treatment, while also examining how rGO affects physiological status, oxidative responses, and specialized metabolic profiles. Plants subjected to different rGO concentrations were evaluated through morphological observations, physiological and biochemical measurements, and assessment of oxidative stress responses. Based on the physiological and biochemical screening results, T3 (100 mg L−1 rGO) at 21 d after treatment was selected for the focused multi-omics comparison. Transcriptomic and metabolomic analyses were then performed using stem tissues from CK and T3 plants collected from the same greenhouse experiment. By linking these levels of evidence, this study captured the response of *D.chrysotoxum* to rGO elicitation across physiological, metabolic and transcriptional scales and identified candidate genes and putatively annotated metabolic features associated with the biosynthesis of key active compounds, particularly flavones and terpenoids. These results provide a useful resource for studying secondary metabolism and functional genes in *D.chrysotoxum* and offer a basis for future validation of candidate metabolic responses to exogenous elicitation in medicinal orchids.

## 2 Materials and methods

### 2.1 Plant materials, growth conditions, and sample collection

The experimental materials were obtained from Baoshan, Yunnan Province, China. Three-year-old cultivated seedlings of *D.chrysotoxum Lindl*. with uniform growth and good overall quality were selected. The seedlings were cultivated in a greenhouse at the teaching and experimental base of Sichuan Agricultural University, Chengdu Campus, China (30°05′ N–31°26′ N, 102°54′ E–104°53′ E). The experiment was conducted from November 2025 to January 2026. During cultivation, the greenhouse conditions were maintained at a day/night temperature of approximately 25–28 °C/18–22 °C, relative humidity of approximately 60–75%, a 16/8 h light/dark photoperiod, and a photosynthetic photon flux density (PPFD) of approximately 163 μmol m^−2^ s^−1^.

The experiment was conducted at the pot level. Each treatment contained 20 pots, and each pot contained 3–5 plants. Pots were randomly assigned to greenhouse positions at the beginning of the experiment and were maintained at their assigned positions throughout the treatment period. Treatments were initiated 14 d after planting.

Six rGO treatments were used: CK, T1, T2, T3, T4, and T5, corresponding to 0, 25, 50, 100, 200, and 400 mg L−1 rGO, respectively. rGO was applied by foliar spraying once every 7 d during the 28 d treatment period. Each pot received 100 mL of the corresponding rGO suspension per application, whereas CK pots received 100 mL of reverse-osmosis (RO) water using the same procedure. Physiological and biochemical responses were evaluated at 7, 14, 21, and 28 d after treatment. At each sampling time, three pots were randomly selected from each treatment as three biological replicates, and tissues were collected from plants at the same developmental stage.

The experiment was designed as a dose-time screening followed by a focused multi-omics comparison. Based on the physiological and biochemical screening, particularly the responses observed at 21 d, T3 (100 mg L−1 rGO) was selected for transcriptomic and metabolomic analyses because it showed the most favorable statistically supported physiological and biochemical profile, including enhanced antioxidant enzyme activities, increased polysaccharide accumulation, increased soluble sugar content, and relatively low membrane lipid peroxidation compared with higher-dose treatments.

For the focused multi-omics comparison, stem tissues from CK and T3 plants were collected at 21 d after treatment from the same greenhouse experiment. RNA-seq and untargeted metabolomic analyses were performed using stem tissues collected at this time point, with six biological replicates per group. qRT-PCR validation was also conducted using stem tissues collected at 21 d from the same experimental batch. Physiological and biochemical measurements were performed using the corresponding tissues specified for each assay: leaves were used for chlorophyll and total flavonoid measurements, whereas stems were used for stem total flavonoids, polysaccharides, soluble sugars, RNA-seq, metabolomics, and qRT-PCR analyses. Samples used for RNA-seq and metabolomic analyses were immediately frozen in liquid nitrogen and stored at −80 °C until analysis.

The physiological and biochemical assays, transcriptomic analysis, metabolomic analysis, and qRT-PCR validation were treated as complementary analytical layers. The physiological and biochemical assays were used for dose-time screening and global biochemical assessment, whereas RNA-seq and untargeted metabolomics were used for a focused tissue-matched comparison between CK and T3 stem samples at 21 d. Therefore, the colorimetric assays and LC-MS metabolomics were interpreted as complementary rather than directly equivalent datasets.

### 2.2 Preparation of reduced graphene oxide

Reduced graphene oxide (rGO) was prepared in-house through a two-step procedure, including the preparation of graphene oxide (GO) by a modified Hummers method followed by KOH-assisted hydrothermal reduction.

Briefly, GO was first synthesized from graphite powder using a modified Hummers method. Under magnetic stirring and ice-bath conditions, 230 mL of concentrated sulfuric acid, 10 g of graphite powder, 5 g of sodium nitrate, and 30 g of potassium permanganate were sequentially and slowly added into a 2 L beaker. The mixture was then transferred to an oil bath and stirred at 35 °C for 30 min to obtain mixture A. Subsequently, 200 mL of deionized water was slowly added to mixture A, followed by the addition of another 260 mL of deionized water. The temperature was then increased to 95 °C, and the mixture was stirred for 15 min. After that, 700 mL of warm deionized water was added, and the reaction was continued for 30 min. Finally, 25 mL of hydrogen peroxide was added and allowed to react for 10 min to obtain mixture B. The resulting suspension was repeatedly washed with deionized water until the pH approached neutrality, and the obtained GO was collected and dried for subsequent reduction.

The prepared GO was then reduced to rGO using a KOH-assisted hydrothermal method. Specifically, 0.75 g of GO powder was dispersed in 50 mL of deionized water and ultrasonicated for 2 h to obtain a homogeneous GO suspension. Separately, 50 mL of 4 M KOH solution was prepared. The GO suspension was added to the KOH solution and stirred at 400 rpm for 1 h. The resulting mixture was transferred into a Teflon-lined stainless-steel autoclave and heated at 150 °C for 8 h. After cooling to room temperature, the obtained dark-black rGO material was collected by vacuum filtration. The product was repeatedly washed with deionized water until the pH reached 7, followed by three additional washes with ethanol. Finally, the rGO was dried in a vacuum oven at 120 °C until completely dry.

Before plant treatment, the dried rGO powder was redispersed in reverse-osmosis (RO) water to prepare suspensions at final concentrations of 25, 50, 100, 200, and 400 mg·L^-1^. The suspensions were ultrasonicated before each application to minimize aggregation and ensure homogeneous dispersion. Foliar spraying was performed once every 7 d throughout the 28 d treatment period. Each pot received 100 mL of the corresponding rGO suspension per application, and CK pots were sprayed with 100 mL of RO water using the same procedure. Fresh rGO suspensions were prepared before spraying. The crystal structure was characterized using X-ray diffraction (XRD; D8 ADVANCE Plus, Bruker, Germany). The surface morphology and layer structure were examined using scanning electron microscopy (SEM; TESCAN MAIA 3 LMH, TESCAN, China) and transmission electron microscopy (TEM; Tecnai G2 F20 S-TWIN TMP, FEI, USA). High-resolution transmission electron microscopy (HRTEM; Talos F200X, Thermo Fisher Scientific, USA) was used to analyze the lattice fringe features. Structural characterization was also performed by Raman spectroscopy using a Renishaw inVia Qontor system (UK) with an excitation wavelength of 532 nm.

### 2.3 MDA and antioxidant enzymes measurement

The activities of superoxide dismutase (SOD, BC5165), catalase (CAT, BC0205), and peroxidase (POD, BC0095) were determined using commercial assay kits (Solarbio, Beijing, China, https://www.solarbio.com). All measurements were performed spectrophotometrically, according to the manufacturer’s instructions. Absorbance was recorded using a UV-visible spectrophotometer. MDA content was measured using the thiobarbituric acid (TBA) reaction, and absorbance values of the red reaction product at 450, 532, and 600 nm were used to calculate MDA equivalents (Hodges et al., 1999). The tissue type, sampling time, treatment group, biological replicate number, and analytical role of each assay are summarized in **Supplementary Table 11**.

### 2.4 Total chlorophyll content and soluble sugar

For total chlorophyll determination, 0.05 g of leaf tissue was weighed and placed in a 25 mL glass weighing tube, followed by extraction with 25 mL acetone:ethanol (1:1, v/v). The samples were then incubated in the dark at 40 °C for 12 h, with repeated mixing during the extraction period. Absorbance at 663 and 645 nm was measured using a UV-visible spectrophotometer (Cary 60, Agilent, USA).

The total chlorophyll content was calculated using Equations 1–3, as described by Nakano and Asada (1981):

(1) Ca=12.72 ×  A663−2.59×A645

(2) Cb=22.88×A645−4.67×A663

(3) Ct=Ca+Cb=20.29×A645+8.05×A663

where Ca denotes the chlorophyll a content (mg·L^-1^), Cb denotes the chlorophyll b content (mg·L^-1^), Ct denotes the total chlorophyll content (mg·L^-1^), and A663 and A645 represent the absorbance values at 663 and 645 nm, respectively.

For soluble sugar determination, the *Dendrobium* stem samples were first ground using a freeze grinder (XFSTPRP-CL, Shanghai Jingxin, China). A 0.1 g portion of each sample was extracted with 8 mL of 80% (v/v) ethanol at 80 °C. After centrifugation at 3,000 × *g* for 30 min, the supernatant was collected for subsequent analysis. The total soluble sugar content was determined at 620 nm using the anthrone method (Tian et al., 2019).

### 2.5 Measurement of total flavonoid, polysaccharide, and total phenol contents

The crude polysaccharide content in *D.chrysotoxum* stems was determined using a Crude Polysaccharide Content Assay Kit (BC6165; Solarbio, Beijing, China). Fresh stem tissues were collected, and extracts were prepared for subsequent analyses as described above. The total phenol content was quantified using the Folin-Ciocalteu (FC) method (Abualzulof et al., 2024). For this assay, an appropriate volume of the extract was combined with suitably diluted FC reagent and allowed to react at room temperature for an appropriate period. Na_2_CO_3_ solution was added to induce alkalization and color development. After incubation in the dark until the color stabilized, absorbance was measured at 765 nm. Quantification was performed using a gallic acid standard curve, and the results were expressed as mg GAE·g^-1^ FW. Total flavonoid content (TFC) was determined using an aluminum chloride-based method adapted from Kim et al. (2024), with appropriate modifications according to related reports. Briefly, 200 μL of sample extract was mixed with 1.0 mL of 10% (v/v) Folin-Ciocalteu reagent and incubated at room temperature for 5 min. Then, 800 μL of 7.5% (w/v) Na_2_CO_3_ solution was added, and the reaction mixture was incubated in the dark at room temperature for 30 min. Absorbance was measured at 765 nm. Gallic acid was used as the standard, with a calibration range of 0-100 μg mL−1, and total phenol content was expressed as mg gallic acid equivalent g^−1^ fresh weight (mg GAE g^−1^ FW).

Total flavonoid content was determined using the NaNO_2_-AlCl_3_ colorimetric method. Briefly, 1.0 mL of sample extract was mixed with 0.3 mL of 5% (w/v) NaNO_2_ and incubated for 5 min. Then, 0.3 mL of 10% (w/v) AlCl_3_ was added and incubated for 6 min, followed by the addition of 2.0 mL of 1 mol L^−1^ NaOH. The final volume was adjusted to 10.0 mL with deionized water, and absorbance was measured at 510 nm after thorough mixing. Rutin was used as the standard, with a calibration range of 0-100 μg mL^−1^, and total flavonoid content was expressed as mg rutin equivalent g^−1^ fresh weight (mg RE g^−1^ FW). Reagent blanks were included in all assays, and each biological replicate was analyzed with technical replicates. Mean values were used for statistical analyses.

### 2.6 RNA extraction and library construction

A transcriptomic dataset was generated from the T3 treatment group exposed to 100 mg·L^-1^ rGO, with six biological replicates. Total RNA was isolated from the tissues using TRIzol^®^ reagent, following the manufacturer’s protocol. RNA integrity was evaluated using a 5300 Bioanalyzer (Agilent), whereas RNA quantity was determined using an ND-2000 NanoDrop. Only samples meeting the quality requirements for sequencing library preparation were retained, namely concentration ≥ 20 ng/μL, total RNA > 1 μg, and RIN ≥ 4.5 for standard-volume samples, or concentration ≥ 10 ng/μL, total RNA > 10 ng, and RIN > 6.5 for microvolume samples. The reference gene source was *D.chrysotoxum*, and read mapping was performed against the reference genome version GCA_019925795.1 obtained from the NCBI database (https://www.ncbi.nlm.nih.gov/datasets/genome/GCA_019925795.1/).

Library preparation and sequencing, including RNA purification and reverse transcription, were performed by Shanghai Mabo Biopharmaceutical Co., Ltd. (Shanghai, China) according to the manufacturer’s instructions (Illumina, San Diego, California). Transcriptome libraries were constructed using the SMART-Seq V4 Ultra-Low Input RNA Sequencing Kit (Clontech) with 10 ng of total RNA as input. Reverse transcription was first performed to synthesize the first-strand cDNA. Poly(A)-containing RNA, mainly mRNA, was reverse-transcribed using oligo(dT) primers. Owing to the activity of the reverse transcriptase (MMLVRT) used in the system, three cytosine residues were added to the 3’ end of the cDNA strand. Template-switching oligonucleotide (TSO) primers were subsequently used to generate the cDNA strand, thereby replacing the RNA complementary to one of the cDNA strands. The resulting cDNA was amplified by PCR to the nanogram scale. Fragmentation and adapter addition were achieved using a modified high-activity Tn5 transposase, which inserted adapters at both ends of the cDNA. After the final round of PCR amplification, the libraries were sequenced. Following quantification with Qubit 4.0, sequencing was performed on the NovaSeq X Plus platform (PE150) using the NovaSeq kit(Illumina, USA).

### 2.7 Quality control and Read mapping

Default parameter processing in fastp was applied to raw paired-end reads for adapter trimming and quality filtering (Chen et al., 2018). The filtered clean reads were subsequently aligned to the reference genome in directional mode with HISAT2 (Kim et al., 2015), after which sample-wise transcript assembly was performed in reference-based mode using StringTie (Pertea et al., 2015).

### 2.8 Transcriptomic analysis

The DEGs between the two sample groups were evaluated based on transcript abundance and gene-level quantification. For each transcript, expression values were normalized as transcripts per million (TPM), and gene abundance was estimated using RSEM. Statistical identification of DEGs was performed using DESeq2 (Love et al., 2014). Genes were regarded as significantly differentially expressed when they met the criteria of |log_2_FC| ≥ 1 with FDR < 0.05 in DESeq2. To determine the functional characteristics represented by the DEG set, enrichment analyses of GO terms and KEGG pathways were conducted against the complete transcriptomic background. Entries satisfying a Bonferroni-corrected P-value < 0.05 were considered significantly enriched. GO enrichment was performed using Goatools, while KEGG pathway analysis was conducted using Python scipy. All transcriptomic analyses were performed using the Majorbio Cloud platform (https://cloud.majorbio.com/) (Han et al., 2024).

For transcriptome-metabolome correlation network analysis, candidate genes, transcription factors, and putatively annotated metabolomic features related to flavonoid and terpenoid metabolism were selected based on FDR-controlled DEG results, FDR-controlled differential-metabolite results, and pathway annotation. Gene expression values and metabolite abundances from the same 12 stem samples (CK1-CK6 and T3_1-T3_6) were used for pairwise correlation analysis. Spearman rank correlation coefficients were calculated for all gene-metabolite pairs, and the corresponding *P* values were adjusted using the Benjamini-Hochberg FDR method. Correlations with |ρ| ≥ 0.80 and FDR < 0.05 were retained for network visualization. Networks were visualized in R using ggplot2. Because the analysis was based on two treatment groups, the resulting associations were interpreted as treatment-associated co-variation rather than evidence of direct regulation or enzymatic function.

### 2.9 Metabolomic analysis

Untargeted metabolomic profiling was performed using stem tissues from CK and T3 plants collected at 21 d after treatment, with six biological replicates per group. For each sample, 100 mg of frozen tissue powder was transferred into a 2 mL centrifuge tube containing a 6 mm grinding bead. Metabolites were extracted with 800 μL of methanol:water (4:1, v/v) extraction solvent containing internal standards, including L-2-chlorophenylalanine (0.02 mg mL^−1^). The samples were homogenized for 6 min at −10 °C and 50 Hz, followed by ultrasonic extraction for 30 min at 5 °C and 40 kHz. The extracts were incubated at −20 °C for 30 min and then centrifuged at 13,000 × g for 15 min at 4 °C. The supernatant was transferred to autosampler vials for LC-MS/MS analysis.

To monitor analytical stability and reproducibility, equal volumes of all sample extracts were pooled to generate a quality-control (QC) sample. QC samples were injected periodically after every 5–10 study samples during instrumental analysis. Metabolite detection was performed using an ultra-high-performance liquid chromatography-tandem Fourier transform mass spectrometry system (UHPLC-Q Exactive, Thermo Fisher Scientific, USA) at Majorbio Bio-Pharm Technology Co., Ltd. (Shanghai, China). Chromatographic separation was performed on a BEH C18 column (100 mm × 2.1 mm i.d., 1.7 μm; Waters, USA). Mobile phase A consisted of water/acetonitrile (98:2, v/v) containing 0.1% formic acid, and mobile phase B consisted of acetonitrile containing 0.1% formic acid. The flow rate was 0.40 mL min^−1^, the column temperature was 40 °C, and the injection volume was 3 μL. The complete chromatographic gradient program, including the initial and final mobile-phase composition, washing step, total run time, and re-equilibration conditions, is provided in **Supplementary Table 17**.

Mass spectrometric data were acquired in both positive and negative electrospray ionization modes over an m/z range of 50–1200. The spray voltage was 3500 V in positive mode and −3000 V in negative mode. The sheath gas and auxiliary gas were set to 50 and 13 arb, respectively. The ion source temperature was maintained at 450 °C, and MS/MS spectra were acquired using stepped collision energies of 20, 40, and 60 eV.

Raw LC-MS/MS data were imported into Progenesis QI software (Waters Corporation, Milford, MA, USA) for baseline filtering, peak detection, integration, retention-time correction, and peak alignment, generating a data matrix containing retention time, mass-to-charge ratio, and peak intensity information. Metabolite annotation was performed by matching MS and MS/MS information against the MJDBPM plant metabolite database developed by Majorbio. After merging and deduplicating data matrices generated under positive and negative ion modes, subsequent preprocessing was performed on the Majorbio Cloud platform (https://cloud.majorbio.com/). Preprocessing included filtering missing values according to the 80% rule, replacing missing values with the minimum value, normalizing total peak area, removing variables with a relative standard deviation (RSD) > 30% in QC samples, and applying log10 transformation.

Multivariate statistical analysis was conducted using PCA as the primary unsupervised analysis. PLS-DA was performed as an exploratory supervised visualization using the ropls package in R, and the model was evaluated by seven-fold cross-validation and 200 permutation tests. Because supervised models may overfit high-dimensional metabolomics datasets with limited sample size, VIP values were used only for exploratory ranking and were not used as the primary criterion for differential-feature calling. For univariate differential analysis, Student’s t-test was performed for each metabolomic feature, and *P values* were adjusted using the Benjamini-Hochberg false discovery rate (FDR) method. Features with FDR < 0.05 were considered significantly differential, and fold change and log2FC were reported as effect-size measures. Differential metabolic features with putative annotations were mapped to the KEGG database for pathway annotation, and Fisher’s exact test was used to determine enriched pathways.

### 2.10 qRT-PCR analysis

**Supplementary Table 3** lists the primer sequences used for qRT-PCR validation of the selected candidate genes. Primers were designed using Primer Premier 5.0. For cDNA synthesis, 1 μg of total RNA was processed using the HiScript IV All-in-One Ultra RT SuperMix for qPCR kit (Vazyme, China) according to the manufacturer’s instructions. RT-qPCR measurements were obtained on a LightCycler 480 system (Roche, Switzerland) using the BG0014 SYBR Prime qPCR Set (BioGround Corp, Chongqing, China). Each target gene was analyzed using three independent biological replicates, and each biological replicate was assayed in three technical replicates. Relative transcript abundance was determined using the 2^-ΔΔCt^ method, and the results were expressed as the mean ± standard deviation (SD). qRT-PCR validation was performed using stem tissues collected from CK and T3 plants at 21 d after treatment, corresponding to the same tissue type, sampling time, and experimental batch used for RNA-seq analysis. *RPL7A* was used as the reference gene for normalization because it showed relatively stable Ct values among CK and T3 stem samples in the present empirical qRT-PCR comparison and has been used as an internal control in related Dendrobium studies. We did not perform a formal multi-algorithm reference-gene stability assessment; therefore, the use of *RPL7A* should be considered an empirical normalization choice for the present CK-versus-T3 validation experiment. Primer specificity was confirmed by the presence of a single peak in melting-curve analysis. Relative expression levels were calculated using the 2^−ΔΔCt^ method. Each treatment included three biological replicates, with technical replicates for each qRT-PCR reaction.

### 2.11 Statistical analysis

All statistical computations and figure-related data processing were performed in R software (version 4.3.2). For physiological and biochemical indices, rGO concentration, sampling time, and their interaction were analyzed using two-way ANOVA. The model included treatment concentration (CK and T1-T5), sampling time (7, 14, 21, and 28 d), and the concentration × time interaction as fixed factors. When significant effects were detected, Duncan’s multiple comparison test was used for *post hoc* comparisons. Statistical significance was defined as *p* < 0.05. For the physiological and biochemical dose-time data, an ordinary two-way ANOVA was performed using rGO concentration, sampling time, and their interaction as fixed factors. Each treatment × time combination included three biological replicates. The full ANOVA output is provided in the **Supplementary Material**.

## 3 Results

### 3.1 rGO characterization

As shown in **Supplementary Figure 1**, the successful preparation of rGO was confirmed by its structural and morphological characteristics. In the XRD pattern, the characteristic GO (001) diffraction peak in the low-angle region disappeared, whereas a broadened (002) peak emerged at a higher angle. This shift indicates that the oxygen-containing functional groups and interlayer water were effectively removed during reduction, accompanied by a marked decrease in interlayer spacing and conversion of the original highly oxidized layered structure into disordered graphitized carbon. The broadened (002) peak further suggests low crystallinity and pronounced structural disorder, with graphitized domains remaining only locally ordered over a short range. Observations from electron microscopy were consistent with these structural changes. SEM showed that rGO retained a two-dimensional layered morphology, while clear surface wrinkling, curling, and local restacking were observed. These features reflect stress release and enhanced π–π interactions after the removal of functional groups, which hinders dense stacking while increasing exposed surface area and interfacial abundance. TEM revealed transparent ultrathin carbon sheets with limited layer stacking, and HRTEM images showed lattice fringes of approximately 0.34 nm, corresponding to the (002) interplanar spacing. These results indicate that the reduction led to the formation of locally ordered graphitized regions within rGO. These microstructural characteristics provide rGO with a large specific surface area and active interfaces, which may promote its interaction with plant surfaces or the cellular microenvironment and support its subsequent biological activity.

TEM observations confirmed the presence of rGO particles within plant cells and chloroplast-associated regions, suggesting that rGO can enter cellular microenvironments and contribute to subsequent physiological and metabolic responses (**Supplementary Figure 2**).

### 3.2 Physiological indicators related to the effects of rGO treatment on *D.chrysotoxum*

**Figure 1:**
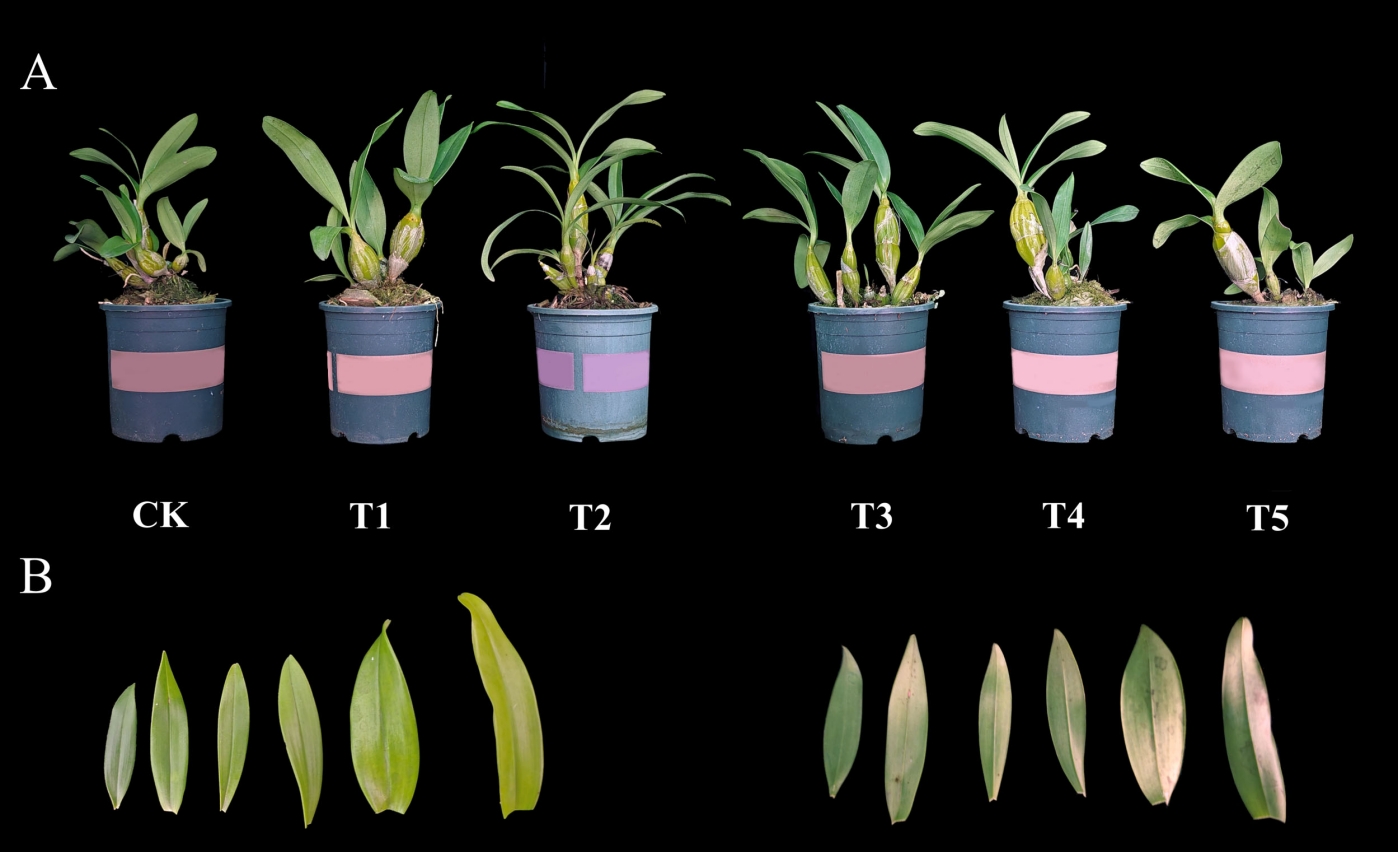
Effects of rGO treatment on *Dendrobium chrysotoxum*. **(A)** Overall morphology of CK and T1–T5 plants at 28 d. **(B)** Leaf morphology of CK and T1–T5 plants at 28 d. The left panel shows the adaxial (front) side, and the right panel shows the abaxial (back) side.

**Figure 2:**
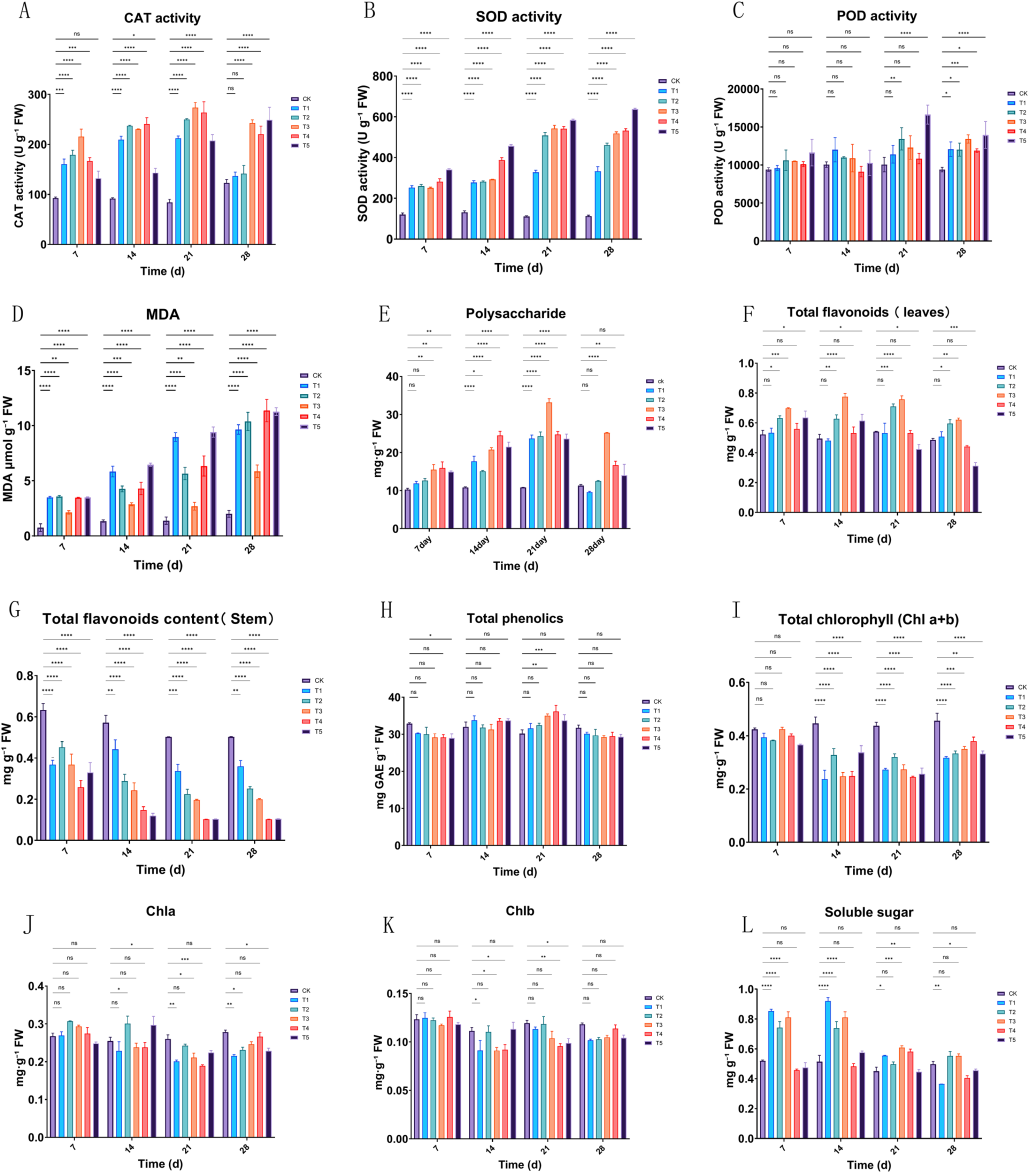
Effects of rGO treatment on physiological and biochemical indicators of *Dendrobium chrysotoxum*. Bar charts show the physiological and biochemical responses of different treatments at each time point: **(A)** catalase (CAT) activity; **(B)** superoxide dismutase (SOD) activity; **(C)** peroxidase (POD) activity; **(D)** malondialdehyde (MDA) content; **(E)** polysaccharide content; **(F)** Total flavonoid content in leaves; **(G)** Total flavonoid content in stems; **(H)** total phenol content; **(I)** total chlorophyll (Chl a+b) content; **(J)** chlorophyll a (Chl a) content; **(K)** chlorophyll b (Chl b) content; **(L)** soluble sugar content. CK denotes the control group, and T1–T5 represent the treatment groups with different rGO concentrations. Data are presented as mean ± SD (n = 3). Statistical significance was determined using two-way ANOVA. ns, not significant; *P < 0.05, **P < 0.01, ***P < 0.001, ****P < 0.0001. The full two-way ANOVA results, numerical dataset, response matrix, and complete statistical output are provided in **Supplementary Tables 7**-**S10**.

*D.chrysotoxum* plants were exposed to 0, 25, 50, 100, 200, and 400 mg·L^-1^ rGO, corresponding to CK and T1–T5, respectively. Samples were collected at 7, 14, 21, and 28 d. Phenotypic indices (e.g., leaf length, leaf width, stem length, and stem diameter) (**Supplementary Table 1**; **Figure 1**) and physiological indicators (**Figure 2**) were then evaluated. By day 28, only limited variation in leaf length, leaf width, stem length, and stem diameter was observed among the treatment groups. Across these indices, the values showed a non-significant tendency to increase from CK to T3 and then decline slightly in T4 and T5, with T3 generally showing the highest numerical values. A similar non-significant pattern was observed for relative growth rates over the 0–28 d period: T1–T4 were generally slightly higher than CK, whereas T5 showed the lowest relative growth rates for all indices and was overall lower than the control. These results indicate that rGO induced non-significant growth-related trends, with low-to-moderate concentrations showing slightly higher numerical values and high concentrations showing slight suppression-related tendencies. Therefore, the selection of T3 was not based on statistically significant growth promotion but on the physiological and biochemical responses described below. To further explore the basis of this response, a range of physiological indicators was measured under both control and rGO treatment conditions, including antioxidant enzymes (CAT, SOD, and POD), membrane lipid peroxidation (MDA), carbohydrate-related traits (polysaccharides and soluble sugars), photosynthetic pigments (chlorophyll), and phenol/flavone-related parameters (total phenols and total flavones) (**Figure 2**).

Compared with CK, all rGO treatments enhanced antioxidant enzyme activities to varying degrees. The CAT activity increased as early as day 7, and the strongest response was observed in T3, where the increase reached approximately 225.17%. The activity peaked on day 21 and remained elevated until day 28. Overall, CAT activity in T1–T4 rose initially and then declined, whereas T5 continued to increase throughout the treatment period (**Figure 2A**). For SOD, the major response occurred at day 21; compared with CK at the same sampling time, T2 and T3 showed increases of 356.4% and 387.8%, respectively. In contrast, T4 and T5 displayed a more sustained upward trend (**Figure 2B**). The POD activity changed only slightly at the early stages but increased markedly between days 21 and 28, with the high-dose treatments showing the strongest response (**Figure 2C**). In parallel with these enzyme responses, the MDA content increased over time. A pronounced increase was detected from day 14 onward in T4 and T5, and high levels persisted through days 21-28, indicating more severe membrane damage under high-dose exposure. In contrast, T3 maintained relatively low MDA levels throughout the experiment, suggesting more effective control of oxidative injury (**Figure 2D**).

Clear temporal changes were also observed in carbohydrate-related traits. The polysaccharide content increased significantly from day 7, rose further between days 14 and 21, and reached its maximum on day 21, with the largest increase occurring in T3 (approximately 206.72%). Even on day 28, T3 remained significantly higher than CK (P < 0.001) (**Figure 2E**). Soluble sugar accumulation was most pronounced between days 7 and 14, with the strongest early increase observed in T1. Significant differences were still present on day 21, whereas the intergroup variation had largely diminished by day 28 (**Figure 2L**).

Phenolic- and flavone-related indices demonstrated evident tissue specificity. In leaves, the Total flavonoid content generally increased first and then declined, with T3 reaching a peak on day 14 that was approximately 56.79% higher than that of the control before falling again by day 28 (**Figure 2F**). In stems, the flavone content remained below that of CK at all time points, and the decline became more pronounced with increasing rGO concentrations (**Figure 2G**). The total phenol content varied only slightly overall, with minor differences on days 7 and 21, and no significant differences were detected among groups on days 14 and 28. Photosynthetic pigments showed an overall reduction during the middle phase of treatment. The total chlorophyll (Chl (a+b)) declined significantly on days 14 and 21, and values in T1–T5 were generally lower than those in CK. A similar overall decrease was observed for both Chl a and Chl b (**Figures 2I–K**).

To further clarify the dose-time response patterns, the physiological and biochemical data were analyzed using a full factorial two-way ANOVA model with rGO concentration, sampling time, and their interaction as fixed factors (**Supplementary Table 7**). Significant concentration × time interactions were detected for most indicators, including CAT, SOD, POD, MDA, polysaccharide, total flavone, chlorophyll components, and soluble sugar, indicating that the physiological effects of rGO depended on both exposure concentration and sampling stage. To visualize these patterns, relative responses were summarized as log2 fold changes compared with CK at the same sampling time (**Supplementary Figure 3**). The heatmap showed that moderate rGO exposure, particularly T3 at 21 d, was associated with strong antioxidant enzyme responses and polysaccharide accumulation, while MDA accumulation was lower than that observed under higher-dose treatments during the main response period. Interaction plots for selected primary variables further showed the replicate-level temporal trajectories across treatments (**Supplementary Figure 4**). The full numerical dataset, relative-response matrix, and complete statistical output are provided in **Supplementary Tables 8**-**S10**.

Taken together, the dose-time physiological and biochemical screening indicated that T3 (100 mg·L^-1^ rGO) represented the most appropriate treatment for subsequent molecular and metabolic analyses based primarily on statistically supported physiological and biochemical responses. Compared with the higher-dose treatments, T3 maintained relatively low MDA levels while showing enhanced antioxidant enzyme activities and the strongest increase in polysaccharide accumulation, especially at 21 d after treatment.

### 3.3 Transcriptomic analysis of effects of rGO treatment on *D.chrysotoxum*

**Figure 3:**
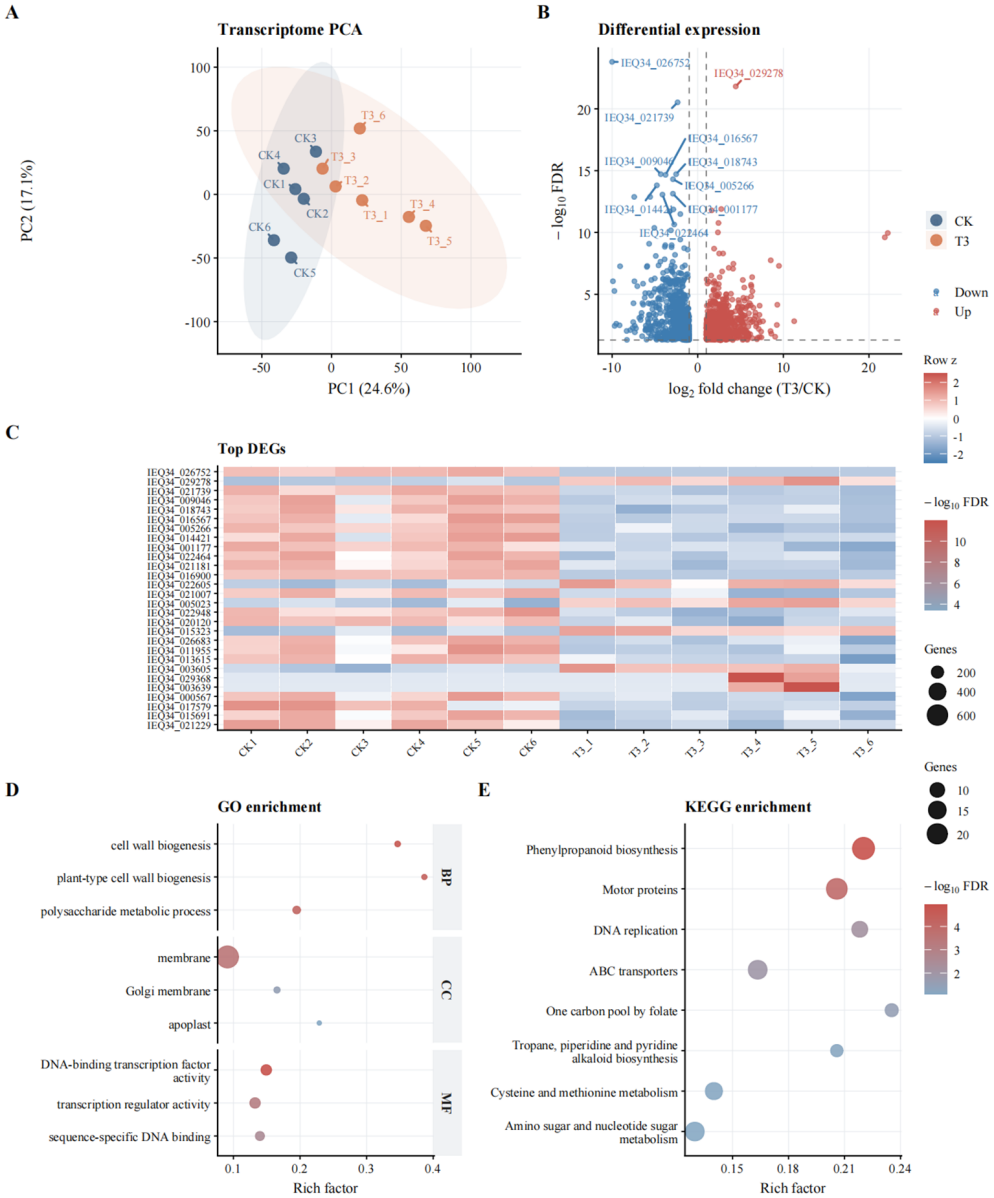
Transcriptomic differential expression and functional enrichment analyses between T3 and CK in *Dendrobium chrysotoxum*. **(A)** PCA score plot. **(B)** Volcano plot showing the distribution of DEGs (x-axis: log_2_FC; y-axis: -log_10_(adjusted *P* value); red, upregulated; blue, downregulated; gray, not significant; representative genes are labeled). **(C)** Hierarchical clustering heatmap of DEGs. **(D)** GO enrichment analysis showing significantly enriched terms in three categories: biological process (BP), molecular function (MF), and cellular component (CC). Color indicates -log_10_(adjusted *P* value). **(E)** KEGG enrichment bubble plot (x-axis: rich factor; dot size indicates the number of genes; color indicates the adjusted *P* value).

This study generated transcriptomic data from 12 samples, yielding 75.09 Gb of clean reads in total. For each sample, the clean-data output was no less than 5.74 Gb, and the Q30 value remained at or above 95.93%, indicating high sequencing quality. The alignment of the clean reads to the designated reference genome produced mapping rates ranging from 90.65% to 92.86%, further supporting the reliability of our transcriptomic dataset. Principal component analysis (PCA) showed a clear separation between the two sample groups along PC1 (42.68%) and PC2 (12.95%), whereas replicates within each group clustered tightly, indicating excellent stability and reproducibility of the treatment response (**Figure 3A**). Using the thresholds of |log_2_FoldChange| > 1 and FDR/*P*adj < 0.05, a total of 1,804 DEGs were identified, including 975 upregulated and 829 downregulated genes (**Figure 3B**). The DEG heatmap further showed stable expression patterns across biological replicates and clear clustering between the compared groups. On the clustering tree, the samples were separated into two major branches, with CK and T3 replicates clustering independently. At the gene level, one cluster was generally upregulated in T3 and downregulated in CK, whereas another cluster showed the opposite pattern, remaining higher in CK and lower in T3. (**Figure 3C**). These clustering patterns can provide the basis for subsequent functional enrichment analysis aimed at identifying pathways and candidate genes associated with the rGO response.

GO enrichment analysis was performed to examine the potential functions represented by the DEGs. The enriched terms were primarily associated with cell wall organization and carbohydrate-related metabolism. Significant enrichment was detected for cell wall biogenesis (GO:0042546; 35 genes), plant-type cell wall biogenesis (GO:0009832; 29 genes), plant-type cell wall tissue or biogenesis (GO:0071669; 30 genes), cell wall tissue or biogenesis (GO:0071554; 55 genes), polysaccharide metabolism (GO:0005976; 70 genes), and carbohydrate metabolism (GO:0005975; 125 genes) (Padj < 0.05, **Figure 3D**).

KEGG analysis further revealed that the DEGs were significantly enriched in multiple pathways related to defense responses and specialized metabolism. As illustrated in **Figure 3E**, phenylpropanoid biosynthesis was among the most significantly enriched pathways. Significant enrichment was also observed for several typical stress- and immunity-related pathways, including plant hormone signal transduction, MAPK signaling pathway-plant, plant-pathogen interaction, and the transport-related pathway ABC transporters. In addition, pathways associated with carbon allocation, such as starch and sucrose metabolism, amino sugar and nucleotide sugar metabolism, and one-carbon pool by folate, were significantly enriched. Several pathways linked to secondary metabolism and quality formation were also represented, including flavonoid biosynthesis, biosynthesis of various plant secondary metabolites, and tropane, piperidine and pyridine alkaloid biosynthesis (**Figure 3E**).

### 3.4 Metabolomic analysis of the effects of rGO treatment on *D.chrysotoxum*

**Figure 4:**
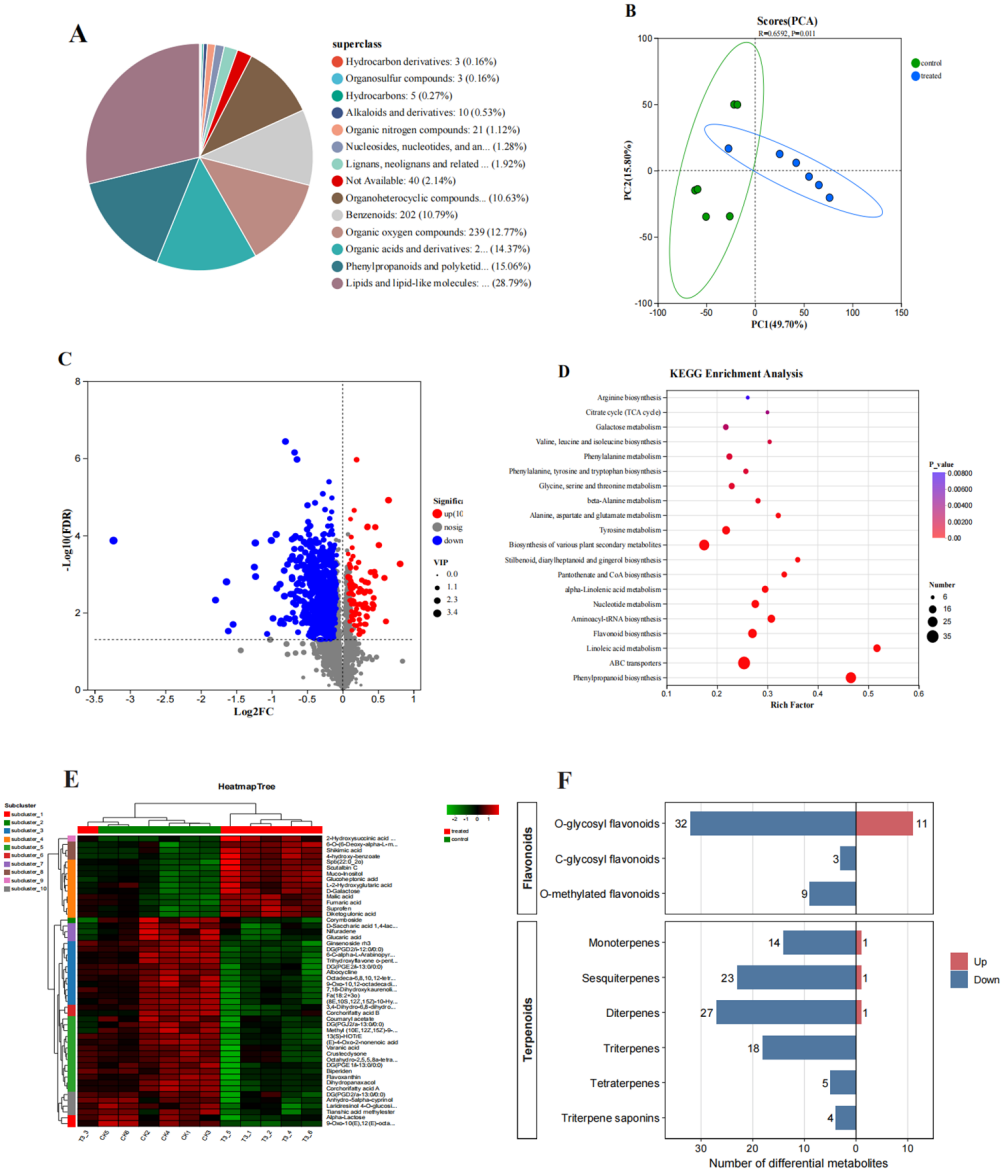
Metabolomic differences in *Dendrobium chrysotoxum* following T3 treatment. **(A)** Chemical classification of detected metabolomic features. **(B)** PCA score plot of CK and T3 samples. **(C)** Volcano plot of FDR-controlled differential metabolomic features between T3 and CK. **(D)** KEGG enrichment bubble plot of differential metabolomic features. **(E)** Hierarchical clustering heatmap of differential metabolomic features. **(F)** Distribution of upregulated and downregulated flavonoid- and terpenoid-related features under rGO treatment. Metabolite names represent putative annotations rather than confirmed compound identifications.

Untargeted metabolomic analysis was conducted for the CK and T3 groups to characterize the metabolic response of *D.chrysotoxum* to rGO treatment. In total, 3,339 metabolomic features were detected and putatively annotated. Notably, lipids and lipid-like molecules constituted the largest proportion (28.79%), followed by phenylpropanoids and polyketides (15.06%), organic acids and derivatives (14.37%), organic oxygen compounds (12.77%), benzenoids (10.79%), and organoheterocyclic compounds (10.63%) (**Figure 4A**).

Pronounced differences in the overall metabolic pattern were observed between CK and T3. In PCA, PC1 and PC2 accounted for 49.70% and 15.80% of the total variance, respectively, and a significant difference between the two groups was detected (R = 0.6592, P = 0.011; **Figure 4B**). A similar separation was observed in the exploratory PLS-DA model, in which Components 1 and 2 explained 46.2% and 7.27% of the variation, respectively. The model showed R2X = 0.614, R2Y = 0.934, and Q2 = 0.761, with 200 permutation tests indicating *P* < 0.05 for both R2Y and Q2 (**Supplementary Figure 5**). Because this supervised model was used only for exploratory visualization, differential metabolomic features were defined using FDR-controlled univariate analysis rather than PLS-DA VIP values alone. These results showed that rGO treatment was accompanied by a substantial shift in the metabolomic profile of *D.chrysotoxum*, while the close clustering of biological replicates supported the stability and reproducibility of the dataset (**Figure 4B**).

The KEGG enrichment further indicated that the differential metabolites were mainly distributed in pathways related to phenylpropanoid biosynthesis, flavone biosynthesis, linoleic acid metabolism, α-linolenic acid metabolism, ABC transporters, pantothenic acid and coenzyme A biosynthesis, stilbene/diarylheptanoids/gingerol biosynthesis, and the biosynthesis of various plant secondary metabolites (**Figure 4D**). Taken together, these pathway-level changes suggest that the metabolic response to rGO involves not only basal metabolic processes but also pathways associated with lipid signaling and secondary metabolic reprogramming in *D.chrysotoxum*.

Using FDR-controlled univariate analysis as the primary criterion, 1,005 metabolomic features were detected as significantly differential between T3 and CK (FDR < 0.05), including 235 upregulated and 770 downregulated features. Fold change and log2FC were reported as effect-size measures. PLS-DA (**Supplementary Figure 5**) was retained as an exploratory supervised visualization and was not used as the primary evidence for differential-feature calling. This distribution indicates that the metabolic response to rGO was largely characterized by a downward shift. The assessment of the representative active constituents of D.chrysotoxum demonstrated that not all characteristic metabolites responded similarly. The dendrobine, increased to 1.25-fold of the control level. In contrast, features putatively annotated as the bibenzyl and phenanthrene derivatives erianin, gigantol, coelonin, and batatasin I decreased to 56.59%, 54.32%, 85.90%, and 88.91% of the control, respectively. A similar selective pattern was identified among flavonoid-related metabolites, where tricin 7-xyloside increased by 1.34-fold, and quercetin 7-glucoside decreased to 0.88-fold of the control. Overall, rGO did not uniformly affect the characteristic active components of *D.chrysotoxum* but altered their accumulation in a selective manner (**Figure 4E**).

A broader pattern was observed when the differential metabolomic features were grouped according to their putative structural classes based on database annotation. Among the features putatively assigned to terpenoid-related classes, most showed lower abundance in T3 than in CK. Specifically, 137 of the 143 differential terpenoid-related features were decreased, whereas 6 were increased. This tendency was observed across features putatively classified as monoterpenes, sesquiterpenes, diterpenes, triterpenes, and tetraterpenes, suggesting that rGO treatment was associated with a broad reduction in detected terpenoid-related features rather than changes confined to a single subclass.

Flavonoid-related features also showed an overall decreasing tendency but displayed a more heterogeneous response pattern. Among the 90 differential flavonoid-related features, 76 were decreased, whereas the direction of change differed among putative modification types. Features putatively assigned to O-methylated flavonoids were generally decreased, whereas flavone-glycoside-related features showed selective changes. For example, features putatively annotated as neocarthamin, prunin, and tricin 7-xyloside were increased in T3. Further grouping suggested that C-flavone-glycoside-related features were mostly decreased, whereas some O-flavone-glycoside-related features were increased. These patterns suggest that rGO treatment may be associated with class- and modification-specific remodeling of flavonoid-related features, rather than a uniform activation or suppression of flavonoid metabolism. (**Figure 4F**).

### 3.5 Integrated transcriptomic and metabolomic analyses

**Figure 5:**
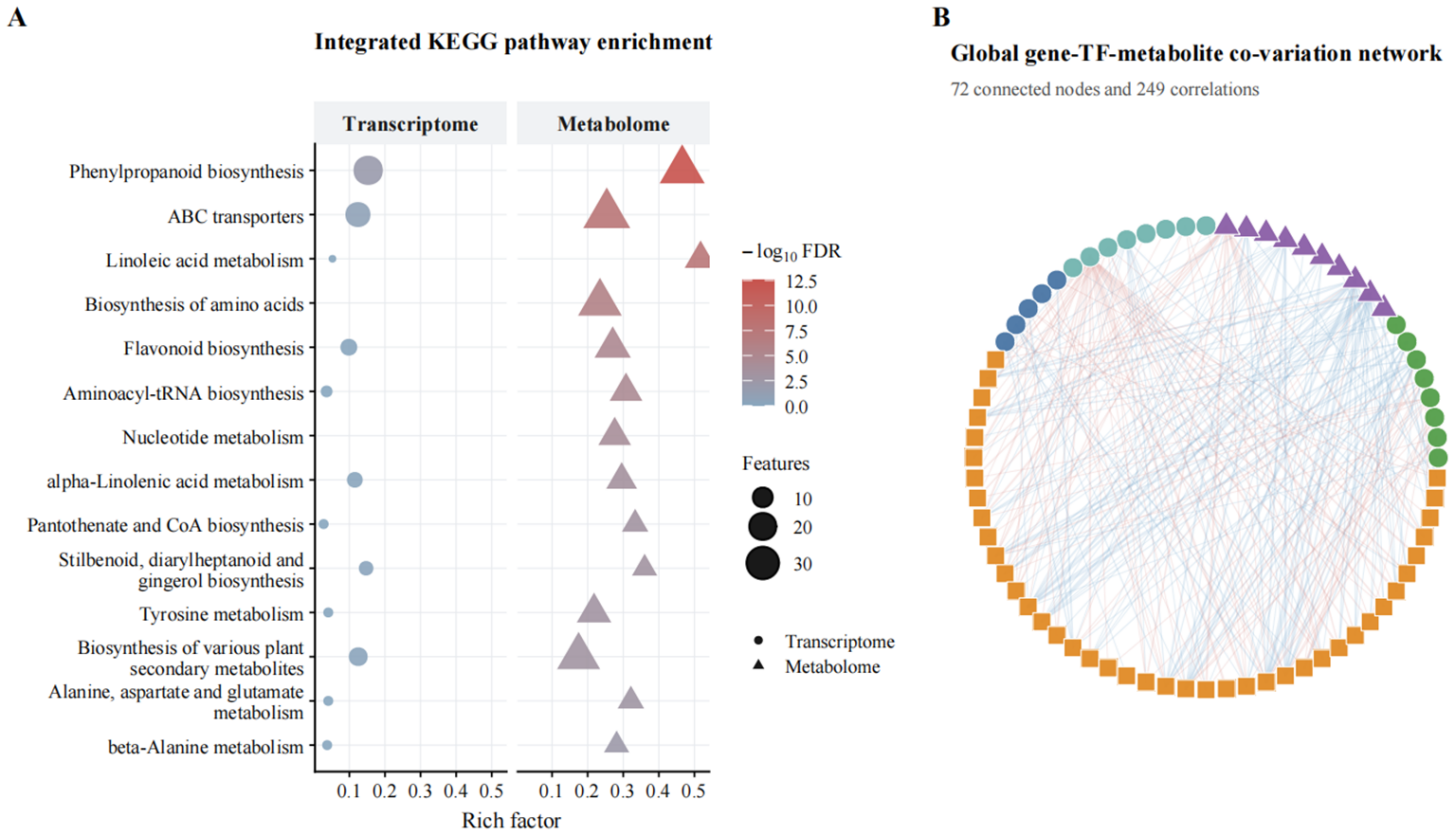
KEGG enrichment and integrative network analysis of DEGs and differential metabolites in T3 in *Dendrobium chrysotoxum*. **(A)** KEGG enrichment analysis of differential features from the transcriptome and metabolome (bubble size indicates the number of features, and color indicates the P value). **(B)** Integrated correlation network. Yellow triangles, green circles, purple circles, and hollow squares represent transcription factors, structural genes, metabolites, and metabolite classes, respectively. Red, blue, and gray edges indicate positive correlations, negative correlations, and category-specific relationships, respectively.

Integrated transcriptomic and metabolomic analyses were performed to trace the global molecular response to rGO treatment. After multiple correction filtering, only five KEGG pathways remained significantly enriched in the T3-versus-CK comparison (FDR < 0.05): α-linolenic acid metabolism (map00592), phenylpropanoid biosynthesis (map00940), flavonoid biosynthesis (map00941), diterpene biosynthesis (map00904), and the biosynthesis of various plant secondary metabolites (map00999) (**Figure 5A**). Overall, the differential metabolic changes were mainly concentrated in precursor metabolism related to lipid signaling and secondary metabolic biosynthetic pathways associated with defense and quality formation. These results suggest that rGO treatment may trigger signal transduction and defense-related metabolic reprogramming through α-linolenic acid metabolism and further promote coordinated responses in the phenylpropanoid-flavonoid and terpenoid pathways. To clarify the potential global relationships between differential metabolites and candidate genes, correlation analysis was conducted using the major differential metabolites and pathway-related genes identified by screening, and a gene-metabolite correlation network was constructed to explore treatment-associated co-variation. The network contained 46 candidate gene/transcription-factor nodes, 43 putatively annotated metabolite nodes, and 249 retained correlations (|ρ|≥ 0.80 and FDR < 0.05; **Supplementary Table 15**).

The putatively annotated metabolite nodes were classified into 11 functional categories, including flavones, terpenoids, alkaloid biomarkers, and bibenzyl/phenanthrene biomarkers.

The distribution of significant correlations was highly uneven across the network and was concentrated mainly in the flavone and terpenoid modules. The terpenoid module comprised 28 significantly correlated genes, 22 metabolites, and 152 significant correlations, whereas the flavone module included 23 genes, 14 metabolites, and 74 significant correlations. In contrast, the benzyl/phenanthrene and alkaloid biomarker modules were smaller, with only 19 and 4 significant correlations, respectively. Within the flavone branch, the broadest association ranges were observed for O-flavone glycosides and O-methylated flavones, which involved 18 significantly correlated genes with 31 significant correlations and 18 significantly correlated genes with 33 significant correlations, respectively. Within the terpenoid branch, the largest correlation scales were found for sesquiterpenes, diterpenes, and monoterpenes, involving 20, 17, and 16 significantly correlated genes, respectively. These network-level patterns support the view that flavone and terpenoid metabolism represent the principal directions of rGO-induced metabolic reprogramming (**Figure 5B**). Therefore, subsequent analyses focused on these pathways and their associated candidate regulatory modules.

### 3.6 Effects of rGO treatment on genes related to the flavonoid biosynthetic pathway

**Figure 6:**
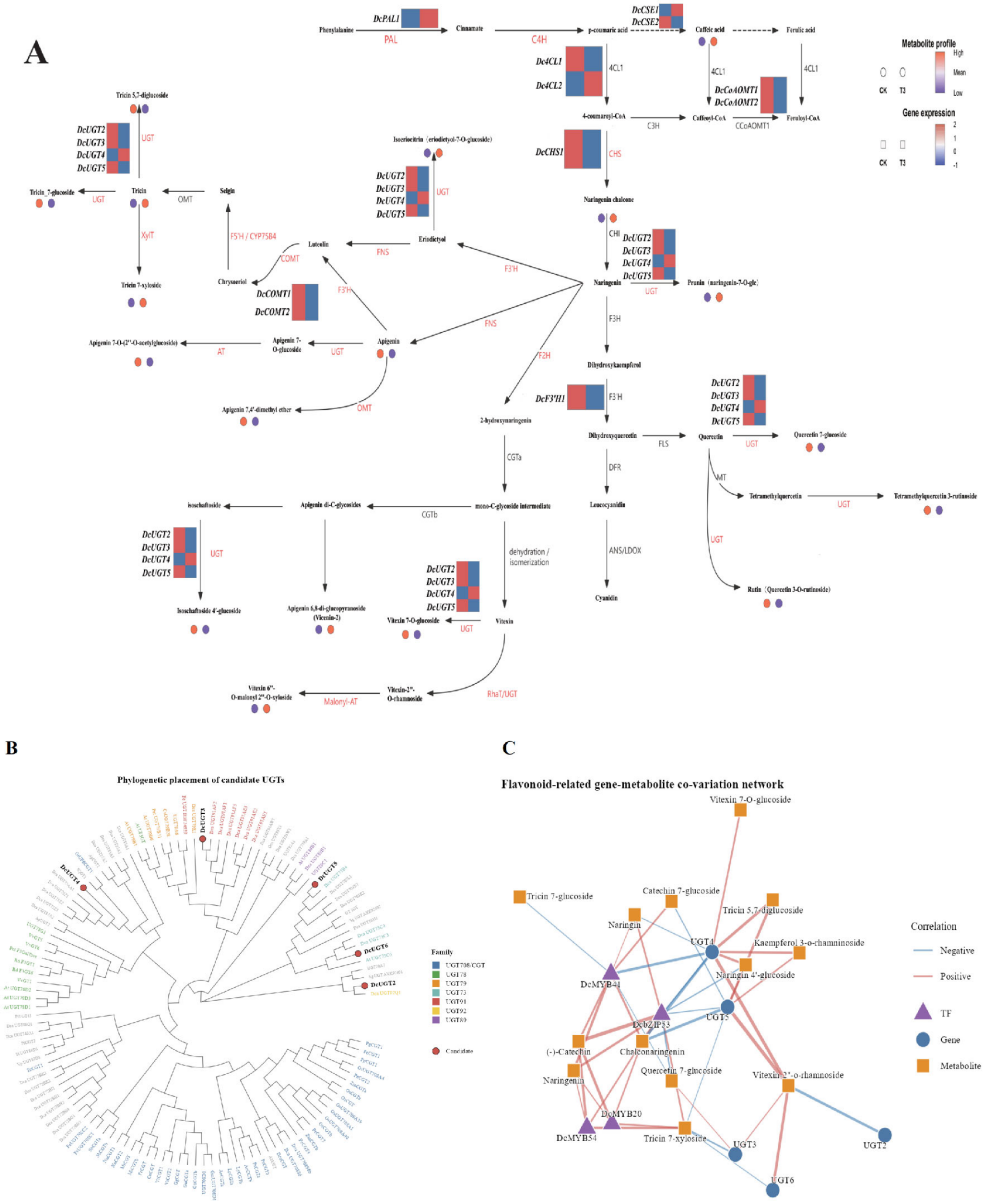
Flavonoid biosynthesis reprogramming, regulatory network analysis, and phylogenetic characterization of candidate UGTs in *Dendrobium chrysotoxum* following rGO treatment. **(A)** Integrated diagram of the flavonoid biosynthetic pathway, showing changes in metabolite abundance and expression patterns of candidate DEGs following rGO treatment. Circles represent metabolite abundance, with color gradients indicating relative accumulation levels. Rectangles represent the gene expression levels, with color gradients indicating relative expression. **(B)** Phylogenetic tree of candidate UGTs and reported plant UGT family members. Colors denote different UGT clades, and candidate genes are labeled. **(C)** Flavonoid correlation network. Orange circles represent transcription factors, blue squares represent structural genes, and green triangles represent metabolites. Edge color indicates Spearman correlation (red, positive; blue, negative), and edge width indicates correlation strength.

Mapping of putatively annotated differential metabolic features and DEGs onto the flavone biosynthetic pathway highlighted 16 candidate genes and 4 transcription factors. The responsive changes induced by rGO were concentrated mainly in three parts of the pathway rather than being evenly distributed: the upstream phenylpropanoid segment, flavone backbone formation, and terminal modification. Within these pathway regions, *DcPAL1* (IEQ34_014562), *DcCSE1* (IEQ34_020258), *Dc4CL2* (IEQ34_020859), and *DcUGT4* (IEQ34_007990) were upregulated in T3, whereas *DcCSE2* (IEQ34_029442), *Dc4CL1* (IEQ34_009679), *DcCoAOMT1* (IEQ34_000651), *DcCHS1* (IEQ34_020495), *DcF3’H1* (IEQ34_021268), *DcUGT3* (IEQ34_001059), *DcUGT4* (IEQ34_004642), and *DcUGT5* (IEQ34_006597) were downregulated (**Figure 6A**).

Several metabolites previously reported in *Dendrobium catenatum*, including naringenin chalcone, vitexin malonyl-xyloside, quercetin 7-O-glucoside, and kaempferol 3-O-rhamninoside, are regarded as important flavone-related compounds in *Dendrobium*. Some of these metabolites have also been associated with antioxidant, anti-inflammatory, antiviral, and potential antitumor activities, particularly naringenin chalcone, quercetin 7-O-glucoside, and quercetin/kaempferol- and vitexin-derived compounds (Lei et al., 2018; Sun et al., 2024; Zhao et al., 2021). In the present analysis, *DcUGT5* showed significant treatment-associated correlations with features putatively annotated as tricin 5,7-diglucoside, naringin 4’-glucoside, and naringenin chalcone, whereas its phylogenetic placement was closely linked to UGT sequences with reported 7-O- and 3-O-glycosylation functions. This combination of correlation and phylogenetic evidence nominates *DcUGT5* as a candidate gene for future functional validation.

A comparable pattern was observed for DcUGT3, but with a different metabolite group. Features putatively annotated as tricin 7-glucoside, vitexin 7-O-glucoside, and quercetin 7-glucoside also appeared in the correlation network in this pathway. Previous studies have associated tricin 7-glucoside with neuroprotective and anti-inflammatory activities (Yu et al., 2024; Zhu et al., 2024), whereas quercetin 7-O-glucoside has been linked to antioxidant effects and the inhibition of influenza A and B viruses (Gansukh et al., 2016). Against this metabolite background, phylogenetic analysis placed *DcUGT3* close to GTs with glucose-glycosylation functions, making it a reasonable candidate for this metabolite set.

### 3.7 Effects of rGO treatment on terpenoid biosynthesis pathway-related genes

**Figure 7:**
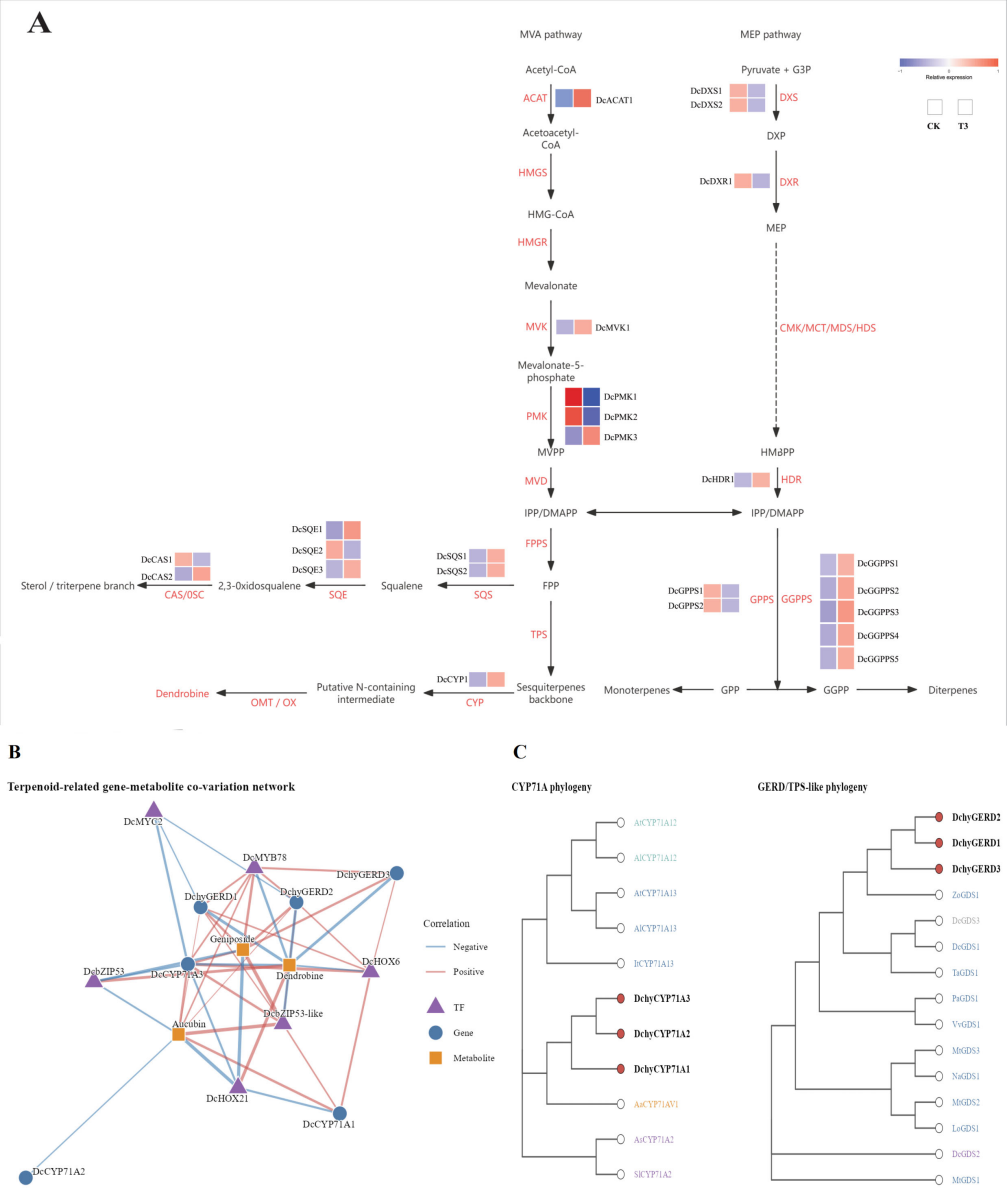
Terpenoid biosynthesis reprogramming, regulatory network analysis, and phylogenetic characterization of candidate genes in *Dendrobium chrysotoxum* following rGO treatment. **(A)** Terpenoid biosynthetic pathway showing changes in metabolite abundance and expression patterns of candidate DEGs following rGO treatment. Circles represent metabolite abundance, with color gradients indicating relative accumulation levels. Squares represent gene expression levels, with color gradients indicating relative expression. **(B)** Terpenoid correlation network. Orange circles represent transcription factors, blue squares represent structural genes, and green triangles represent metabolites. Edge color indicates Spearman correlation (red, positive; blue, negative), and edge width indicates correlation strength. **(C)** Phylogenetic analysis of terpenoid-related candidate genes. The upper panel shows the phylogenetic relationships between candidate CYP71A proteins and their reported homologs. The lower panel shows the relationships between candidate GDS/GERD proteins and TPS family members. Different colors denote distinct clades, and candidate genes are labeled.

Within the terpenoid pathway, seven candidate genes and two transcription factors were highlighted, showing a pattern comparable to that observed for flavones. The integration of pathway-level information indicated that terpenoid metabolism was markedly affected by rGO treatment. At the precursor-supply level, *DcACAT1* (IEQ34_003495), *DcDXS1* (IEQ34_022827), *DcDXS2* (IEQ34_022825), and *DcDXR1* (IEQ34_002697) were identified as key rate-limiting enzyme genes in the MVA and MEP pathways (**Figure 7A**). However, their responses were not uniform. *DcACAT1* was upregulated following rGO treatment, whereas *DcDXS1*, *DcDXS2*, and *DcDXR1* were downregulated. Clear responses were also detected in the downstream branches, including the sterol/triterpene and sesquiterpene-framework branches. In T3, the upregulation was observed for *DcMVK1* (IEQ34_006288), *DcPMK3* (IEQ34_017750), *DcHDR1* (IEQ34_004744), *DcSQS1* (IEQ34_005275), *DcSQS2* (IEQ34_008818), *DcSQE1* (IEQ34_016388), *DcSQE3* (IEQ34_011772), *DcCAS2* (IEQ34_008964), and *DcGGPPS1-5* (IEQ34_003293, IEQ34_004854, IEQ34_007952, IEQ34_029696, IEQ34_020575), whereas *DcPMK1* (IEQ34_001217), *DcPMK2* (IEQ34_028442), *DcSQE2* (IEQ34_017062), *DcCAS1* (IEQ34_010214), *DcGPPS1* (IEQ34_015413), *DcGPPS2* (IEQ34_015659), and *DcCYP1* (IEQ34_008437) were downregulated. Because dendrobine is an important Dendrobium alkaloid, the terpenoid-related correlation network was examined with an emphasis on the feature putatively annotated as dendrobine. This analysis showed that *DchyGERD1* (IEQ34_013738), *DchyGERD2* (IEQ34_028480), *DchyGERD3* (IEQ34_013391), and *DcCYP71A3* (IEQ34_009279) were significantly correlated with dendrobine, with the strongest correlations observed for *DchyGERD1*, *DchyGERD2*, and *DchyGERD3*. In parallel, *DcHOX21* (IEQ34_015458), *DcHOX6* (IEQ34_020024), DcMYB78 (IEQ34_004880), DcbZIP53-like (IEQ34_011612), DcbZIP53 (IEQ34_001334), and DcMYC2 (IEQ34_014014) may represent candidate transcription factors associated with dendrobine-related terpenoid variation. (**Figure 7B**). Phylogenetic evidence further supported the identity of these candidates. *DchyCYP71A1*, *DchyCYP71A2*, and *DchyCYP71A3* clustered closely with previously reported homologous proteins of the CYP71A family (**Figure 7C**). CYP71A is a subfamily of plant cytochrome P450 monooxygenases that performs oxidative modifications after TPS/GERD-mediated formation of the terpenoid framework, most commonly hydroxylation, followed by oxidation, epoxidation, and even framework rearrangement. In contrast, *DchyGERD1* and *DchyGERD2* clustered within the GDS-related branch and were clearly separated from other TPS-class proteins. Because germacrene D synthase is a key TPS-class enzyme in sesquiterpene biosynthesis and mainly catalyzes the conversion of FPP into the germacrene D-type framework, this phylogenetic position may support the annotation of these genes as candidate genes related to terpenoid biosynthesis. .

### 3.8 qRT-PCR analysis of candidate genes

To verify the reliability of the transcriptomic results, 10 candidate genes, namely *DcUGT2, DcUGT3, DcUGT4, DcTOGT1*, DcMYB20, DcbHLH130, *DcGERD1, DcGERD2*, *DcCYP71A1*, and *DcCYP71A2*, were selected for RT-qPCR analysis. Expression heatmaps for these genes were generated based on transcriptomic data (**Supplementary Figure 6A**). Overall, the RT-qPCR results showed expression patterns consistent with those obtained from transcriptome sequencing. Compared with CK, *DcUGT2*, DcMYB20, and *DcCYP71A2* were significantly upregulated in T3, whereas *DcUGT3*, *DcUGT4*, *DcTOGT1*, DcbHLH130, *DcGERD1*, *DcGERD2*, and *DcCYP71A1* were significantly downregulated.

## 4 Discussion

As exogenous nano-elicitors, nanomaterials can induce stress-related signaling and metabolic reprogramming without the need for genetic modification, which can offer a potential strategy for targeted regulation of active constituents in medicinal plants. To our knowledge, this study is the first to evaluate the effects of rGO treatment on the physiological and biochemical characteristics and metabolic profiles of *D.chrysotoxum*. In addition, candidate transcript-metabolite associations related to flavonoid and terpenoid metabolism in this species were examined using integrated transcriptomic and metabolomic analyses.

### 4.1 Effects of rGO treatment on the physiological and biochemical indicators of *D.chrysotoxum*

The biological effects of nanomaterials on plants are generally concentration-dependent and often follow a low-dose stimulatory but high-dose inhibitory pattern (Yang et al., 2022). Such responses have also been reported for graphene-based materials. In addition, low concentrations of GO promoted plant height and leaf area in Trifolium repens during short-term exposure, whereas high concentrations negatively affected growth and chlorophyll accumulation (Zhao et al., 2022). Similarly, rGO and GO have been reported to inhibit the growth of Brassica napus L. by reducing chlorophyll content and interfering with photosynthesis (Xiao et al., 2022). A comparable trend was observed in the present study, although the growth-related differences were not statistically significant. Low-to-moderate rGO treatments showed only non-significant positive growth-related trends, whereas higher concentrations were associated with mild dwarfing and leaf yellowing in D. chrysotoxum.

**Figure 8:**
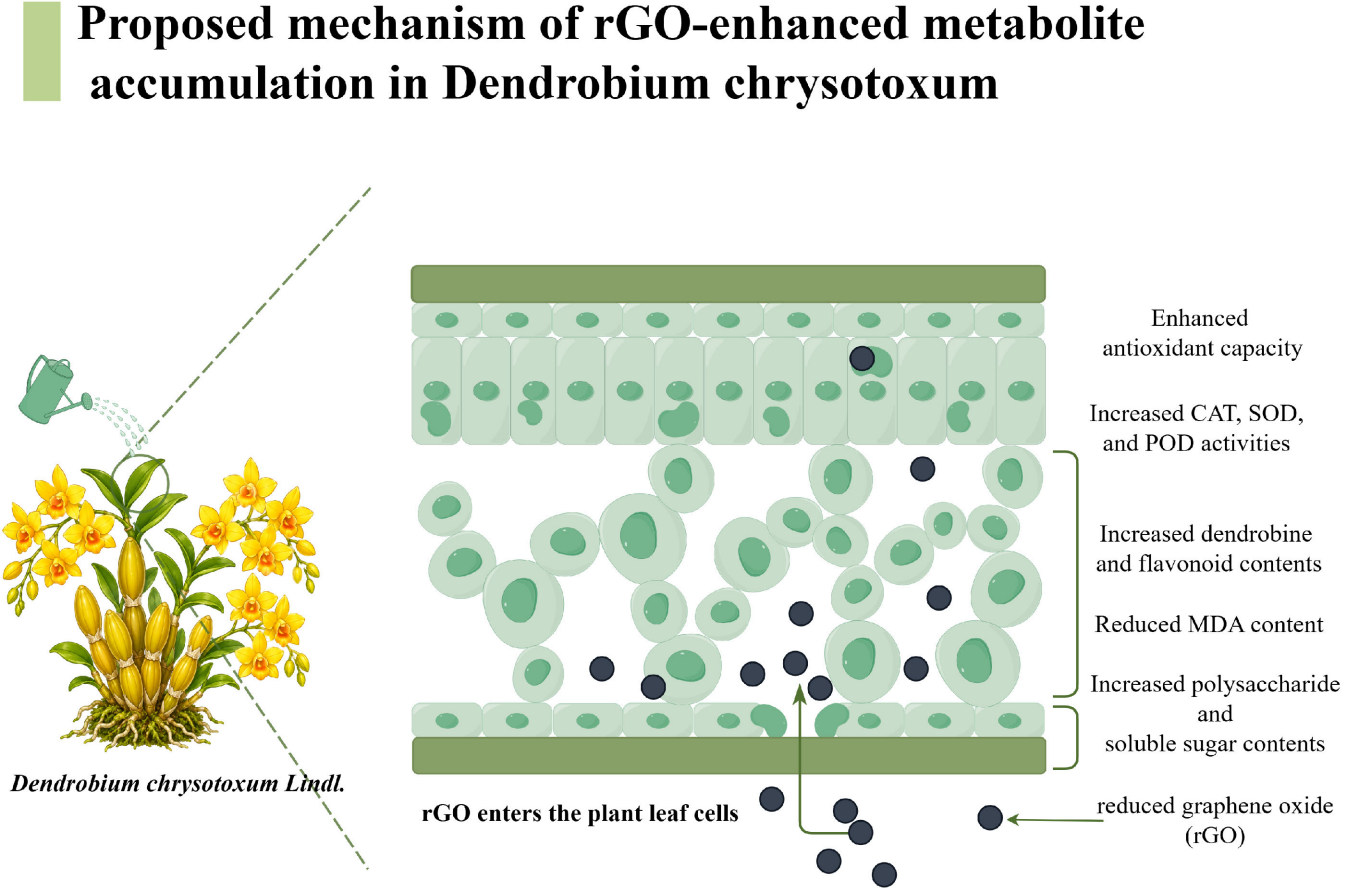
Proposed working model of rGO-associated physiological and metabolic responses in *Dendrobium chrysotoxum* seedlings. Foliar application of reduced graphene oxide (rGO) may enter leaf tissues and trigger dose-dependent physiological and metabolic responses in D. chrysotoxum. Under moderate rGO treatment, antioxidant enzyme activities, including CAT, SOD, and POD, were enhanced, while polysaccharide and soluble sugar accumulation increased and MDA remained relatively lower than that under higher-dose treatments. Integrated transcriptomic and metabolomic analyses further suggested that rGO treatment was associated with changes in redox homeostasis, carbon allocation, flavonoid-related metabolism, and putatively annotated dendrobine-related metabolic features. This model summarizes the treatment-associated responses observed in the present study and provides candidate hypotheses for future mechanistic validation(by figdraw. zcom).

Previous studies have shown that rGO can damage root cells and disturb hormone homeostasis (Li et al., 2018). Under high-concentration exposure, severe electrolyte leakage in red spinach and cabbage has been linked to the inhibition of root growth, indicating oxidative stress and plasma membrane injury (Begum et al., 2011). Furthermore, recent work has highlighted the potential of nanomaterials that regulate ROS homeostasis to enhance crop stress tolerance (Giraldo, 2019). Carboxyl-modified graphene oxide possesses intrinsic peroxidase-like activity (Song et al., 2010). Moreover, GO can function as a potential nanozyme in sorghum by modulating ROS levels, thereby influencing jasmonic acid biosynthesis and insect resistance responses (Shi et al., 2025). Taken together, these findings suggest that the biological effects of rGO may arise from both nanozyme-like activity and nanomaterial-induced stress. Under moderate exposure, the activation of the intrinsic antioxidant system may promote an effective defense response, whereas excessive exposure may disrupt this balance and intensify lipid peroxidation and cellular injury. The observed changes in CAT, POD, SOD, and MDA support this interpretation, with MDA showing a U-shaped response across treatments. Flavonoids and other phenolic compounds are important non-enzymatic antioxidants in plants. Their roles in stress adaptation include ROS scavenging, buffering of photooxidative damage, and participation in hormone-defense signaling interactions. As leaves are a major site of both photosynthesis and ROS generation, the increase in leaf flavone accumulation during the middle stage of treatment may have contributed to buffering photooxidative stress and strengthening non-enzymatic antioxidant capacity. In this study, total flavonoid content increased in leaves during the middle stage of treatment but declined overall in stems, whereas total phenol content changed only slightly. These results suggest that rGO induced tissue-specific changes in non-enzymatic antioxidant metabolism. A proposed working model integrating the treatment-associated physiological and metabolic responses is presented in **Figure 8**.

It should be noted that the global colorimetric assays and the untargeted metabolomic analysis represent complementary but not fully equivalent analytical layers. The leaf flavone data cannot be directly used to explain the LC-MS metabolomic profile, because untargeted metabolomics was performed using stem tissues from CK and T3 plants collected at 21 d after treatment. Therefore, the increase in leaf total flavonoid content was interpreted as an independent physiological response related to leaf antioxidant capacity. In the tissue-matched comparison, however, the decrease in stem total flavonoid content was broadly consistent with the stem metabolomic profile, in which most differential flavonoids were down-regulated in T3. These included features putatively annotated as genkwanin, hispiduloside, neocarlinoside, chrysin 6-C-glucoside 8-C-arabinoside, quercetin 7-glucoside, and several apigenin- and kaempferol-derived glycosides. These metabolites may represent candidate compounds contributing to the reduced Total flavonoid signal in stems.

Nevertheless, several flavonoids were up-regulated in T3, indicating that rGO did not uniformly suppress flavonoid metabolism but instead remodeled the flavonoid profile in a compound-specific manner. In addition, total phenol content was retained as a global phenolic-equivalent biochemical indicator rather than being treated as a direct validation of any single LC-MS metabolite class. This interpretation avoids overextending the relationship between colorimetric measurements and untargeted metabolomics while still highlighting their complementary evidence for rGO-induced metabolic adjustment.

### 4.2 Effects of rGO treatment on flavone synthesis pathway of *D.chrysotoxum*

Flavones are the key active components of *D.chrysotoxum*. High Total flavonoid levels in *D.chrysotoxum* have been associated with good *in vitro* antioxidant activity of its extracts (Xu et al., 2025). The antioxidant capacity of *D.chrysotoxum* flavone extracts is closely associated with flavone content, suggesting that flavones may constitute an essential molecular basis for the health-promoting and pharmacological functions of this species (Hu et al., 2023). In the present study, multiple putatively annotated flavonoid features showed differential abundance under rGO treatment, and distinct response patterns were observed between O-glycosylation and C-glycosylation. O-glycosyltransferases have been identified in multiple plant species, including *Isatis indigotica* (Chen et al., 2021), *Arabidopsis thaliana* (George Thompson et al., 2017), *Clitoria ternatea* (Hiromoto et al., 2015), and *Zea mays* (Bidart et al., 2022). UGT-mediated glycosylation can reduce the toxicity of receptor molecules while enhancing their stability and solubility, thereby helping plants maintain a more controlled and safer antioxidant molecule pool under stress conditions (Behr et al., 2020). Glycosylation is one of the most widespread modification patterns of secondary metabolites (Huang et al., 2015).

Quercetin 7-O-glucoside (Q7G), naringenin chalcone, and catechin 7-glucoside (C7G) are of particular interest. As a glycosylation product of flavones, Q7G is associated with plant defense and stress responses and exhibits strong antioxidant activity (Kim et al., 2022). Recent studies have shown that naringenin chalcone possesses anti-inflammatory and anti-allergic activities (Escribano-Ferrer et al., 2019). C7G is mainly associated with antioxidant activity, free radical scavenging, cell protection, and anti-inflammatory effects (Baek et al., 2011; Kim et al., 2010; Kook et al., 2015). In the present study, rGO treatment had significant effects, regardless of concentration, and influenced the accumulation of flavone glycosides. The correlation network suggested treatment-associated co-variation involving *DcUGT3*, DcMYB41, DcbZIP53, and flavonoid-related features. Phylogenetic analysis indicated that the homologous genes of *DcUGT3* belonged to the glycoside glycosyltransferase (GGT) class. *DcUGT3* was also associated with vitexin-2”-O-rhamnoside. It was speculated that the function of *DcUGT3* could be similar to that of the GGT family and that it could perform secondary modifications on 6C-glycosylation substrates. Moreover, *DcUGT3* may be considered a candidate for future studies of O-site modification of 6C glucose. According to the phylogenetic tree, the homologous genes of *DcUGT*5 and *DcUGT6* primarily mediate 7-O glycosylation. Both were associated with C7G and DcbZIP53. Homologous genes of *DcUGT4* primarily have C-glycosylation functions. *DcUGT4* was associated with naringin 4’-glucoside and tricin 5,7-diglucoside, as well as with DcMYB41 and DcbZIP53. MYB41 can regulate the expression of PAL and DFR (Jin et al., 2024), and DcbZIP53 can directly regulate the expression of *RcUGT27* to modulate kaempferol 3-O-rutinoside (K3R) biosynthesis (Shi et al., 2026). In the present study, DcbZIP53 may simultaneously participate in the regulation of *DcUGT5* and *DcUGT6*, whereas DcMYB41 and DcbZIP53 may jointly participate in the *DcUGT3*-related flavone glycosylation process. However, no published reports directly describe the interaction between MYB41 and bZIP53. MYB41 and bZIP53 may jointly participate in and synergistically influence downstream processes, providing a basis for subsequent experimental validation.

### 4.3 Effects of rGO treatment on terpenoid synthesis pathway of *D.chrysotoxum*

Among *Dendrobium* species, sesquiterpene alkaloids, represented by dendrobine, are regarded as characteristic active components and possess various pharmacological activities, including neuroprotective, anti-inflammatory, antitumor, and antiviral effects (Mou et al., 2021; Tan et al., 2023). In the present study, multiple terpenoid-related features were detected as differentially abundant under rGO treatment, especially monoterpenes, sesquiterpenes, diterpenes, triterpenes, and tetraterpenes. Dendrobine biosynthesis is generally thought to involve MVA/MEP-derived IPP and FPP-dependent sesquiterpene framework formation, although the specific cyclases and downstream modification steps remain incompletely resolved (Wang et al., 2020; Gong et al., 2022). The correlation network showed that this dendrobine-annotated feature was significantly correlated with *DchyGERD1* (IEQ34_013738), *DchyGERD2* (IEQ34_028480), *DchyGERD3* (IEQ34_013391), and *DcCYP71A3* (IEQ34_009279). The phylogenetic tree indicated that *DchyGERD1–3* were homologous genes belonging to the GDS/GERD class of enzymes related to sesquiterpene framework construction. In addition, *DchyGERD1–3* showed positive correlations with DcMYB78, DcHOX6, and DcbZIP53-like, while showing a negative correlation trend with DcMYC2. The transcription factor (TF) families involved in dendrobine synthesis regulation include AP2/ERF, MYB, WRKY, bHLH, bZIP, and NAC, among which bHLH factors, especially MYC2, have the strongest supporting evidence (Chen et al., 2019; Gui et al., 2026). In Ginkgo, MYC2 activates *GbGGPPS* and positively regulates terpenoid biosynthesis (Zheng et al., 2024). In Catharanthus roseus (L.) G. Don, MYC2 also acts synergistically with GBF/bZIP-like factors to regulate indole alkaloid biosynthesis (Sui et al., 2018). Studies on Panax saponins have indicated that MYB78 positively regulates the expression of FPS and SQE (Yang et al., 2025). *DchyGERD1–3* may be associated with dendrobine-related metabolic variation under rGO treatment, and MYC2 and MYB78 may be associated with rGO-responsive dendrobine-related metabolic variation, but their regulatory roles require functional validation in *D.chrysotoxum*.

Iridoid glycosides are common defensive metabolites in plants and exhibit anti-inflammatory, antioxidant, and neuroprotective properties. Ecologically, they are often associated with plant adaptation to herbivores, pathogens, and external stress (Biere et al., 2004; Thabet et al., 2022). In the present study, *DcCYP71A1* (IEQ34_008437) and *DcCYP71A3* (IEQ34_009279) were significantly correlated with aucubin. *DchyGERD2* (IEQ34_028480), *DchyGERD1* (IEQ34_013738), *DchyGERD3* (IEQ34_013391), and *DcCYP71A3* (IEQ34_009279) were significantly correlated with geniposide. In the phylogenetic tree, the homologous genes of DchyCYP71A1 and DchyCYP71A3 are involved in the continuous oxidation of terpenoids and defense-related metabolic transformation (Chen et al., 2024). *DcCYP71A1* and *DcCYP71A3* are candidate genes for future validation of related terpenoid or iridoid-glycoside responses in *D.chrysotoxum*. DcHOX6 (IEQ34_020024), DcbZIP53-like (IEQ34_011612), DcHOX21 (IEQ34_015458), and the genes mentioned above were significantly correlated. In *Dendrobium catenatum*, the bZIP family plays an important role in drought- and stress-related responses (Wang et al., 2023). In *Dendrobium*, the HD-ZIP family is mainly associated with stress-related processes (Huang et al., 2021). rGO may exert similar stress effects on plants, causing metabolite changes in *Dendrobium*.

## 5 Conclusions

This study characterized the concentration-dependent effects of rGO treatment on growth-related traits, physiological status, antioxidant responses, and specialized metabolic profiles in *D.chrysotoxum* seedlings by integrating physiological measurements, transcriptomic analysis, and untargeted metabolomics. Moderate rGO treatment showed non-significant favorable growth-related trends and was associated with increased CAT, SOD, and POD activities, higher polysaccharide and soluble sugar levels, and relatively low membrane lipid peroxidation compared with higher-dose treatments. In contrast, high-concentration rGO treatment resulted in slight dwarfing, leaf yellowing, and increased MDA accumulation after prolonged exposure, indicating that the physiological response to rGO depends strongly on concentration and exposure duration.

Multi-omics analysis indicated that rGO treatment was associated with changes in carbohydrate metabolism, phenylpropanoid metabolism, flavonoid-related metabolism, terpenoid-related metabolism, plant hormone signal transduction, and MAPK signaling. The metabolomic responses were mixed: flavonoid-related features showed divergent changes among glycosylated and methylated derivatives, whereas most terpenoid-associated features were reduced. Although features putatively annotated as dendrobine-related metabolites showed increased abundance, several other characteristic putatively annotated compounds decreased. Therefore, the present results support treatment-associated physiological and metabolomic changes, but they do not demonstrate an overall improvement in medicinal quality.

By integrating phylogenetic analysis, transcriptome-metabolome correlation analysis, and qRT-PCR validation, several UGT, GERD, CYP71A, and transcription-factor genes were prioritized as candidates associated with rGO-responsive metabolic variation. These candidate associations provide hypotheses for future studies but do not establish direct regulatory or enzymatic relationships. Further targeted metabolite quantification, authentic-standard validation, bioactivity assays, enzyme assays, genetic experiments, and safety evaluation will be required to determine whether rGO treatment can be used to improve specific medicinal traits in *D.chrysotoxum*.

## Data Availability

The RNA-seq raw data generated in this study have been deposited in the NCBI Sequence Read Archive under BioProject accession number PRJNA1454189 (https://www.ncbi.nlm.nih.gov/bioproject/PRJNA1454189). The metabolomics datasets and other supporting data are included in this article and its **Supplementary Material**; Processed RNA-seq data have been deposited in Zenodo under DOI: https://doi.org/10.5281/zenodo.21093500. The raw UHPLC-MS/MS metabolomics data have been deposited in OMIX under accession number OMIX018231 (https://ngdc.cncb.ac.cn/omix/release/OMIX018231). The processed metabolomics feature matrix, differential metabolomics table, sample metadata, and supporting data are provided in the **Supplementary Material**.
