# Supplementary material for: Reduced graphene oxide modulates antioxidant responses and specialized metabolism in the medicinal orchid Dendrobium chrysotoxum: an integrated transcriptomic and metabolomic perspective
Source: Front Plant Sci. 2026 Jul 21;17:1912555. doi: 10.3389/fpls.2026.1912555 (PMC13433189; doi:10.3389/fpls.2026.1912555)
Supplement: Supplementary file 1 [file SupplementaryFile1.docx]

Fig. S1. (a) XRD pattern of rGO; (b) SEM image of rGO showing its wrinkled and undulating two-dimensional sheet-like morphology; (c) low-magnification TEM image of rGO; (d) high-resolution TEM (HRTEM) image of rGO.

Fig. S2. TEM observation of cellular ultrastructure in rGO-treated *Dendrobium chrysotoxum* stem tissues.
(a) Low-magnification TEM image showing the cell wall, chloroplast, and large vacuole. (b) Higher-magnification TEM image showing rGO-associated electron-dense regions near chloroplast ultrastructure. Scale bars: 5 μm in (a) and 500 nm in (b).

**Fig. S3. Heatmap of relative physiological and biochemical responses of Dendrobium chrysotoxum under rGO treatment. Values represent log2 fold changes relative to CK at the same sampling time based on treatment means (n = 3). Red and blue indicate increases and decreases relative to CK, respectively.**

**Fig. S4. Interaction plots of selected physiological and biochemical indicators in Dendrobium chrysotoxum under rGO treatment. (A) Catalase (CAT) activity; (B) superoxide dismutase (SOD) activity; (C) malondialdehyde (MDA) content; (D) polysaccharide content; (E) total flavone content in leaves; (F) total flavone content in stems. Individual points denote biological replicates, and lines with whiskers show the mean ± SD (n = 3). CK denotes the control group, and T1-T5 correspond to 25, 50, 100, 200, and 400 mg L^-1 rGO, respectively.**

**Fig. S5. Exploratory PLS-DA model of the stem metabolomic profiles of Dendrobium chrysotoxum under rGO treatment. (A) PLS-DA score plot showing the separation between CK and T3 samples. Component 1 and Component 2 explained 46.2% and 7.27% of the variation, respectively. (B) Permutation test of the PLS-DA model based on 200 permutations. The model showed R2X = 0.614, R2Y = 0.934, and Q2 = 0.761, with permutation-test results of P < 0.05 for both R2Y and Q2. The PLS-DA model was used as an exploratory visualization, whereas FDR-controlled univariate analysis was used as the primary criterion for differential metabolomic feature screening.**

**Fig.S6. qRT-PCR validation of relative expression levels of candidate genes in CK and T3 in Dendrobium chrysotoxum. Data are presented as mean ± SD. Statistical significance is indicated by asterisks: ***P* < 0.01, ****P* < 0.001, and *****P* < 0.0001.**
